# Toxicological Assessment and Dual Protective Roles of *Ophiocordyceps sinensis* Melanin: Photoprotection and Heavy Metal Detoxification

**DOI:** 10.3390/jof12080613

**Published:** 2026-08-15

**Authors:** Huan Yang, Jingyi Wang, Xiangxin Li, Chuanyong Li, Yiming Wang, Yanli Huo, Dandan Fu, Li He

**Affiliations:** College of Biology and Pharmaceutical Engineering, Lanzhou Jiaotong University, Lanzhou 730070, China; yanghuan001103@163.com (H.Y.); wy129976@gmail.com (J.W.); lixx@mail.lzjtu.cn (X.L.); lichuanyong2001@163.com (C.L.); wym030210@163.com (Y.W.); huoylsw@126.com (Y.H.); fdd02272140@163.com (D.F.)

**Keywords:** *Ophiocordyceps sinensis*, melanin structural characterization, biocompatibility, oxidative stress attenuation, heavy metal biosorption

## Abstract

This research investigated the extraction and purification of melanin from the *Ophiocordyceps sinensis* TZ8-1; characterized its structural properties; and evaluated its safety, photoprotective efficacy, and ability to mitigate heavy metals. The results demonstrated that the spectral features of TZ8-1 melanin were consistent with those of synthetic melanin, confirming its classification as typical eumelanin. The safety assessment indicated that TZ8-1 melanin has relatively low toxicity to four types of normal human cells, and based on the results of acute oral toxicity tests, this substance showed high safety for experimental animals. Photoprotection studies showed that TZ8-1 melanin effectively suppressed reactive oxygen species (ROS) generation and significantly improved cell viability post-ultraviolet B (UVB) exposure. Heavy metal mitigation experiments showed that TZ8-1 melanin reduced malondialdehyde (MDA) levels, increased glutathione (GSH) concentrations, and enhanced cell survival rates in heavy metal-exposed conditions.

## 1. Introduction

Chinese cordyceps is a rare medicinal complex resulting from the infection of bat moth larvae by the *Ophiocordyceps sinensis* (*O. sinensis*), primarily found in high-altitude alpine meadow regions [1,2]. Its distinctive growth environment, characterized by low temperature and intense ultraviolet radiation, imparts notable antioxidant, antitumor, and immunomodulatory properties [3]. In recent years, the secondary metabolites of *O. sinensis* have garnered significant research interest. Among which, melanin, an irregular pigment commonly found in various organisms, has drawn considerable attention due to its diverse chemical structure and extensive biological activity. Melanin was primarily synthesized via the L-3,4-dihydroxyphenylalanine (DOPA) and 1,8-dihydroxy naphthalene (DHN) pathways, resulting in the form of eumelanin and brown melanin. It was recognized for its potential in antioxidation, antibacterial activity, anti-radiation effects, and drug delivery [4,5]. Despite its widespread application in biomedicine, cosmetics, and nanomaterials [6,7], systematic research on melanin derived from *O. sinensis* remains relatively limited, particularly regarding safety and the mechanisms underlying its specific biological activities. While Tong et al. (2023) recently provided foundational insights into its chemical characterization and preliminarily reported its UV-blocking, metal-chelating, and ROS-scavenging capacities [3], a critical gap remains regarding its biosafety profile, which is a prerequisite for translational applications.

Furthermore, melanin demonstrates considerable chemical diversity and functional plasticity within the fungal kingdom. Research indicates that melanin derived from distinct fungal sources such as *Inonotus hispidus* (*Basidiomycota*) or *Aspergillus nidulans* (*Ascomycota*) exhibits variations in elemental composition, polymerization degree, and functional group distribution, which directly influenced their efficacy in antioxidation, metal chelation, and UV shielding [8,9]. For example, fungal melanin was predominantly synthesized via the DOPA or DHN pathways, yielding eumelanin or pheomelanin with conjugated aromatic structures that confer free radical scavenging capacity and photostability [5,10]. Despite their promising applications in biomedical materials (e.g., drug delivery systems) and environmental remediation (e.g., heavy metal adsorption), systematic investigations into melanin from fungi inhabiting unique environments, such as *O. sinensis*, remain scarce. Notably, the molecular mechanisms underlying its structure–activity relationship and safety profile are yet to be elucidated [11].

Thus, beyond corroborating these structural characteristics, this study prioritizes a comprehensive toxicological evaluation—including in vitro cytotoxicity across multiple normal cell lines and in vivo acute oral toxicity assessments compliant with OECD guidelines—to establish the safety boundaries of TZ8-1 melanin. Furthermore, we elucidate the underlying mechanisms driving their photoprotective effects (via apoptosis analysis) and heavy metal detoxification capabilities (via MDA/GSH modulation). This in-depth examination not only expands the repository of natural pigments but also lays a solid foundation for developing novel antioxidants and chelating agents.

## 2. Materials and Methods

### 2.1. Materials and Chemicals

Wild Chinese cordyceps was collected from Daiqian Pasture, Tianzhu County, Wuwei City, Gansu Province, China (approximately 37°14′ N, 102°49′ E), in 2019, and we continued to cultivate it in our laboratory for 15 d. After the spores were ejected, the spores were collected for strain isolation and purification, and the strain TZ8-1 was obtained [12].

### 2.2. TZ8-1 Fermentation, Extraction, and Purification of Melanin

The TZ8-1 strain was inoculated into a milk culture medium comprising 200 mL of milk, 200 g of potato, 20 g of glucose (Sinopharm Chemical Reagent Co., Ltd., Shanghai, China), 1 g of KH_2_PO_4_(Tianjin Hengxing Chemical Reagent Manufacturing Co., Ltd., Tianjin, China), 0.2 g of MgSO_4_·7H_2_O (Xilong Chemical Co., Ltd., Foshan, China), 1 g of yeast powder (Thermo Fisher Scientific China, Shanghai, China), 5 g of hydrolyzed milk protein (Beijing Coolabio Technology Co., Ltd., Beijing, China), 15 g of agar (Beijing Coolabio Technology Co., Ltd., Beijing, China), and complex vitamin B1 tablets (Jiangsu Yabang Aipusen Pharmaceutical Co., Ltd., Jiangsu, China). Subsequently, the mixture was incubated at a constant temperature of 16 °C for 60 d. Afterward, the cultivated TZ8-1 strain was transferred to a liquid medium containing 200 g of potato, 20 g of glucose, 10 g of yeast powder, 5 g of peptone (Beijing Aoboxing Biotechnology Co., Ltd., Beijing, China), 1 g of KH_2_PO_4_, and 0.25 g of MgSO_4_·7H_2_O [3]. The culture was then placed in a constant temperature shaker (HZQ-X500C, Shanghai Yihheng Technology Co., Ltd., Shanghai, China) at 160 rpm and 18 °C for an additional 60 d to facilitate the extraction of melanin from TZ8-1.

Filter the mycelial balls from 200 mL of the fermentation broth. Adjust the pH of the broth to 14 using NaOH (Sinopharm Chemical Reagent Co., Ltd., Shanghai, China), and magnetically stir the mixture at 100 °C for 15 h. Upon completion of stirring, centrifuge the mixture at a centrifugal force of 10,000 g for 30 min, retaining the supernatant. Subsequently, adjust the pH of the supernatant to 2 with HCl solution (Sinopharm Chemical Reagent Co., Ltd., Shanghai, China) and incubate it in a water bath (HH-2, Shandong Hualu Instrument Co., Ltd., Shandong, China) at 100 °C for 12 h. Following this, centrifuge the solution again at a centrifugal force of 10,000 g for 30 min and retain the precipitate. Wash the precipitate 3 to 5 times with distilled water and dry it in a vacuum drying oven (DZF-6050, Shanghai Jinghong Experimental Equipment Co., Ltd., Shanghai, China) to obtain the crude product of TZ8-1 melanin [3].

Dissolve the crude pigment in a 7 mol/L HCl solution and incubate at 100 °C for 3 h. Subsequently, centrifuge the mixture to collect the precipitate and wash it 2 to 3 times with distilled water. Follow this by sequential washing with ethanol (Sinopharm Chemical Reagent Co., Ltd., Shanghai, China), chloroform (Sinopharm Chemical Reagent Co., Ltd., Shanghai, China), and ethyl acetate (Sinopharm Chemical Reagent Co., Ltd., Shanghai, China) solutions, and allow the precipitate to dry at room temperature. Next, dissolve the crude dried melanin in a 2 mol/L NaOH solution and centrifuge at 10,000 g for 15 min. After centrifugation, adjust the pH of the supernatant to 2 using a 2 mol/L HCl solution and incubate in a water bath at 100 °C for 3 h. Once the precipitate forms, centrifuge again at 10,000 g for 15 min to collect it. Repeat the precipitation step three times. Wash the precipitate with distilled water 3 to 5 times until it reaches neutrality, and then freeze-dry it (Scientz-30YG/A, Ningbo Scientz Biotechnology Co., Ltd., Ningbo, China) to obtain purified TZ8-1 melanin [12,13].

### 2.3. TZ8-1 Identification of Melanin

#### 2.3.1. Scanning Electron Microscopy Analysis (SEM)

The morphological structure of TZ8-1 melanin was examined using a field-emission scanning electron microscope (ZEISS Gemini SEM 500). The melanin was coated with gold under vacuum conditions and analyzed with a scanning electron microscope [9].

#### 2.3.2. Elemental Analysis (EA)

The contents of five elements—carbon (C), hydrogen (H), oxygen (O), nitrogen (N), and sulfur (S)—in purified TZ8-1 melanin were analyzed using an organic element analyzer (Elementary UNICUBE, Elementar Analysensysteme GmbH, Hanau, Germany).

#### 2.3.3. Ultraviolet–Visible Spectroscopy Analysis (UV-Vis)

The melanin in TZ8-1 was analyzed using an ultraviolet–visible spectrophotometer (UV-8000, Shanghai Metash Instruments Co., Ltd., China) to identify its characteristic absorption peak and maximum absorption wavelength. A total of 0.5 mg of TZ8-1 melanin was dissolved in 0.1 mol/L NaOH solution. The UV–visible absorption spectrum of TZ8-1 melanin was then scanned in the wavelength range of 200–500 nm, with 0.1 mol/L NaOH solution serving as the blank control [14].

#### 2.3.4. Fourier-Transform Infrared Spectroscopy Analysis (FT-IR)

The purified TZ8-1 melanin powder was finely ground and uniformly mixed with KBr at a mass ratio (1:100). Following this grinding process, the mixture was pressed into tablets. The FT-IR Fourier-transform infrared spectrometer was then employed to scan the samples within the range of 4000–400 cm^−1^, thereby obtaining the infrared spectrum of TZ8-1 melanin [8].

#### 2.3.5. Electron Paramagnetic Resonance Spectroscopy (EPR)

Add 5 mg of dried TZ8-1 melanin to a 5 mm specification nuclear magnetic resonance tube and place it in an electron paramagnetic resonance spectrometer. The EPR parameters include central field at 3362.00 G, sweep width of 100.00 G, receiver gain set at 2.00 × 10^4^, modulation frequency of 100.00 kHz, modulation amplitude at 1.00 G, modulation phase of 0.00 degrees, offset at 0.00%, time constant of 81.92 ms, conversion time set to 50.00 ms, harmonic value of 1, dual detection mode set to phase, microwave attenuation at 40 dB, temperature at 300.00 K, frequency at 41.51%, and signal phase at 29.16% [15].

#### 2.3.6. Pyrolysis Gas Chromatography and Mass Spectrometry Analysis (Py-GC-MS)

Experiments were conducted utilizing the Py-GC-MS technique, employing a CDS 6000 Pyroprobe pyrolysis instrument (CDS Analytical, USA) and a GCMS-TQ8050 NX mass spectrometer (Shimadzu, Japan). The procedures adhered to the methodologies established by Li and Stepien et al. [16,17], with minor modifications. The purified TZ8-1 melanin and synthesized melanin samples were maintained at 0 °C for 0 s in Py Mode, subsequently heated to 500 °C for 20 s, and held at that temperature for an additional 20 s, followed by a cleaning phase at 800 °C for 20 s. The temperatures of the transmission line, valve box, and GC interface were all set to 300 °C, with all heaters activated. The initial temperature of the GC column oven was 50 °C, while the injection port was maintained at 280 °C. The split ratio was 5:1, the linear velocity was 39.4 cm/s, and the column flow rate was 1.18 mL/min. The column temperature program was increased from 50 to 280 °C. The mass spectrometry ion source was set to 200 °C, the interface to 260 °C, the solvent delay to 2 min, the scanning range to *m*/*z* 20–600, the scanning mode to Q3 Scan, and the acquisition time spanned from 2.5 to 26 min [18,19,20].

### 2.4. Cytotoxicity Test

Analysis of cytotoxicity was typically required for extracts from plants and isolated natural products [21,22]. In this study, we focused on evaluating the cytotoxicity of melanin extracted from *O. sinensis* using four cell lines: Normal Human Epidermal Keratinocytes (NHEKs, ATCC, BFN60808806), Human Normal Liver Cells, HL-7702 (LO2, ATCC, BFN608006124), Human Kidney Epithelial Cells (Hkb20, ZYBio, ZY-C6246H), and Normal Colon Mucosa 460 (NCM460, ATCC, BFN608006385) [23,24,25]. Cell culture was conducted with Dulbecco’s Modified Eagle Medium (DMEM) medium supplemented with 10% fetal bovine serum (FBS) for LO2, NCM460, and Hkb20, and with KGN keratinocyte serum-free medium for NHEK. The cells were maintained at 37 °C in a 5% CO_2_ atmosphere, with regular medium changes every 2 to 3 d. Upon reaching 80% confluence, cells in the logarithmic growth phase were subcultured after digestion with 0.25% trypsin ethylenediaminetetraacetic acid (EDTA). Subsequently, the cells were centrifuged at 1000 rpm for 5 min, resuspended, counted, and adjusted to a specific concentration. A 24 h pre-culture period was allowed by inoculating 100 μL of the cell suspension into each well of a 96-well plate. TZ8-1 melanin was dissolved in dimethyl sulfoxide (DMSO) (Sigma-Aldrich, St. Louis, MO, USA; Cat# C6295) to prepare a stock solution at 100 mg/mL; stored at −20 °C; and diluted in a gradient with the culture medium to achieve final concentrations of 0, 6.25, 12.5, 25, 50, 100, and 200 μg/mL. Care was taken to ensure that the DMSO concentration in each well did not exceed 0.1% before use. The previous culture medium was removed, and experimental conditions comprised a blank group (cell-free culture medium), a control group (culture medium with 0.1% DMSO), and an experimental group (culture medium with varying concentrations of melanin). Subsequently, 100 μL of the respective culture medium was added to each well, and the cultures were incubated for 48 h. After this incubation period, 10 μL of Cell Counting Kit-8 (CCK-8; Dojindo Laboratories, Kumamoto, Japan; Cat# CK04) solution was added to each well, thoroughly mixed, and then incubated in darkness for 4 h. The optical density (OD) of each well was assessed at a wavelength of 450 nm using an enzyme-linked immunosorbent assay (ELISA) reader. Each experimental condition was tested using three independent biological replicates (distinct cell passages on different days), with each replicate measured in triplicate (technical repeats). Cell viability was determined using formula (1), a concentration–effect curve was generated, and the half-maximal inhibitory concentration (IC50) was calculated using GraphPad Prism software [26].

(1) $$Cell\;survival\;rate\;=\;\frac{ODExperiment-ODBlank}{ODControl-ODBlank}\times 100\%$$

### 2.5. Acute Oral Toxicity Testing (OECD 423)

#### 2.5.1. Preparation of Experimental Animals

C57 experimental mice (Lanzhou Veterinary Research Institute) aged 8 weeks were housed in the barrier facilities of specific pathogen-free (SPF) animal room of Animal laboratory, Lanzhou Institute of animal husbandry and veterinary medicine, Chinese Academy of Agricultural Sciences. Bedding and feed were procured from Beijing Keao Xieli Feed Co., Ltd., Beijing, China. The mice were kept in controlled conditions at 22 ± 1 °C and 55% relative humidity, acclimating to a 12 h light/dark cycle for a week. They had ad libitum access to water and food. The experimental protocol was approved by the Ethics Committee of the Laboratory Animal Research Center at Lanzhou Institute of animal husbandry and veterinary medicine, Chinese Academy of Agricultural Sciences [27] (Approval Number: 2025-67).

#### 2.5.2. Acute Oral Toxicity Test

According to the experimental procedures established by the Organization for Economic Cooperation and Development (OECD) on 423, acute oral toxicity experiments were conducted on mice [28]. The untreated control group (Group 1) and the 2000 mg/kg treated group (Group 2) each comprised 3 independent biological replicates. For the 5000 mg/kg treated group (Group 3), each individual mouse was treated as an independent biological replicate. The animals were randomly assigned to three groups: the untreated control group (Group 1) and two treated groups (Group 2 and Group 3). Following group assignment, all experimental animals underwent an overnight fasting period. After weighing, treatment administration commenced. Group 1 received only distilled water, while Group 2 was administered a melanin suspension at a dose of 2000 mg/kg, and Group 3 received a melanin suspension at 5000 mg/kg (with each administration not exceeding 2000 mg/kg and a 4 h interval between doses). For the 5000 mg/kg administration, one experimental mouse was treated first. Post-administration, the mice were monitored for mortality. If any mice died, the administration of 5000 mg/kg was halted. If no deaths occurred, the remaining two mice were subsequently treated. After oral administration, the systemic behavior of mice was monitored for 30 min, and then at 1, 2, 4, 8, and 24 h. Daily observations of systemic behavior continued up to the 14th day post-administration [27,29,30].

#### 2.5.3. Blood and Sample Collection

Following the experiment’s conclusion, the animals were euthanized for gross dissection. The experimental mice were anesthetized in a transparent anesthesia induction room with 2–3% isoflurane and 1 L/min of oxygen, and the righted reflex disappeared within about 30–60 s. Subsequently, blood samples were drawn from the heart and divided into EDTA tubes with anticoagulants and non-anticoagulant tubes. The samples were then stored at −20 °C until the analysis parameters were determined. For CO_2_ euthanasia, the gas flow rate should be gradually increased (not exceeding 0.5 KPa) once the mice are unconscious. After ensuring the absence of movement, breathing, or pupillary response, the gas supply should be stopped, and observation should continue for an additional 2 min to confirm death.

After the experimental mice experienced a complete cessation of breathing and heartbeat, their livers, kidneys, and other internal organs were removed and macroscopically examined for abnormalities. The internal organs were then preserved in 10% neutral formalin (4% formaldehyde) for 72 h, followed by standard paraffin embedding for section preparation. The organs were cut into 4–5 μm thick slices and stained with hematoxylin and eosin (H&E) before observation under an optical microscope [31].

#### 2.5.4. Biochemical Analysis

Plasma parameters were evaluated using an automated analyzer (Hitachi 7180 or equivalent; Hitachi High-Technologies Corporation, Tokyo, Japan), predominantly employing enzymatic assays for alanine aminotransferase (ALT), aspartate aminotransferase (AST), alkaline phosphatase (ALP), γ-glutamyl transferase (GGT), total bile acid (TBA), creatine kinase (CK), glucose (GLU), triglycerides (TGs), and amylase (AMY), among other analytes [32].

### 2.6. Research on Light Protection

This research examined the photoprotective properties of TZ8-1 melanin against damage induced by ultraviolet B (UVB) radiation in human epidermal keratinocytes (NHEKs). The evaluated damage encompasses decreased cell viability, heightened reactive oxygen species (ROS) accumulation, and apoptosis. Cell viability was gauged utilizing the CCK-8 technique, intracellular ROS levels were determined using the DCFH-DA fluorescent probe, and apoptosis was assessed via Annexin V-FITC/PI double staining combined with flow cytometry [33]. The shielding effects of TZ8-1 melanin pre-exposure against UVB triggered harm were comprehensively examined.

#### 2.6.1. Cell Culture

NHEK cells were cultured in serum-free KGN keratinocyte medium at 37 °C in a 5% CO_2_ environment. The medium was refreshed every 2 to 3 d, and passaging occurred at 80% to 90% cell confluence. Cells in the logarithmic growth phase were harvested using 0.25% trypsin–EDTA (25200056; Gibco, Thermo Fisher Scientific, Waltham, MA, USA), centrifuged, and resuspended in fresh culture medium for cell counting. For the CCK-8 assay, the cell concentration was adjusted to 5 × 10^3^ cells per well in black 96-well plates with a final volume of 100 μL per well. In the ROS assay, the cell concentration was set at 1 × 10^5^ cells per dish in culture dishes. As for the apoptosis assay, the cell concentration was set at 2 × 10^5^ cells per well in 6-well plates with a volume of 2 mL. Subsequently, the cells were incubated and cultured for 24 h until reaching full adherence and entering the logarithmic growth phase [34].

#### 2.6.2. Drug Administration and UVB Irradiation

The experimental groups were organized as follows. (1) Blank group: Culture medium only, with no cells present. (2) Blank control group: Cells present without any treatment. (3) Model group (UVB): Cells present, receiving only UVB irradiation without any medication. (4) Normal control group (VC): Cells present, receiving UVB irradiation with the addition of vitamin C (VC) (1043003; Sigma-Aldrich, St. Louis, MO, USA). (5) Drug group (UVB + melanin): Cells treated with melanin at concentrations of 0, 6.25, 12.5, 25, 50, 100, and 200 µg/mL following UVB irradiation. The old culture medium was discarded, and fresh culture medium containing the corresponding concentrations of drugs or VC was added to each group. Pre-treatment was conducted for 2 h. The UVB dose was calibrated using a radiometer, delivering an irradiation of 30 mJ/cm^2^. Subsequently, the cells were returned to the incubator and incubated in the dark for 24 h prior to detection [35].

#### 2.6.3. Cell Survival Rate

Cell survival rates were evaluated utilizing the CCK-8 technique [36]. After culturing, 10 μL of CCK-8 solution was gently mixed into each well and incubated in darkness for 4 h. The absorbance (OD value) was measured at 450 nm using a microplate reader (ELx808; BioTek Instruments, Winooski, VT, USA). All experiments were independently repeated three times (*n* = 3 biological replicates), with each replicate measured in triplicate. Cell survival rates were calculated according to formula (1). The objective of this study was to assess the impact of UVB exposure and pre-treatment with drugs on cell viability.

#### 2.6.4. Determination of Reactive Oxygen Species Production (ROS)

Intracellular reactive oxygen species (ROS) levels were detected using the 2′,7′-Dichlorodihydrofluorescein diacetate (DCFH-DA) fluorescence probe method [36]. The DCFH-DA probe was diluted to a final concentration of 10 μM in serum-free medium. Subsequently, 1 mL of the diluted probe was added to each dish and incubated in a 37 °C cell incubator in the dark for 20 to 30 min. This incubation allowed the probe to permeate the cells and be oxidized by ROS, resulting in the formation of the green fluorescent product DCF. After incubation, the cells were washed three times with serum-free medium. To further assess nuclear morphology, Hoechst dye was added and incubated for 10 to 15 min, followed by a wash with PBS (10010023; Thermo Fisher Scientific, Waltham, MA, USA). In this procedure, DCFH-DA (S0034S; Beyotime Biotechnology, Shanghai, China) served as a green fluorescent probe (excitation wavelength, Ex = 488 nm; emission wavelength, Em = 525 nm) to indicate intracellular ROS levels, while Hoechst dye (C1022; Beyotime Biotechnology, Shanghai, China) functioned as a blue fluorescent probe (excitation wavelength, Ex = 405 nm; emission wavelength, Em = 461 nm) for nuclear staining, thereby facilitating the observation of morphological changes in the cell nucleus.

#### 2.6.5. Apoptosis Assay

Apoptosis detection was conducted using the Annexin V-fluorescein isothiocyanate/propidium iodide (V-FITC/PI) double-staining method in conjunction with flow cytometry [37]. Cells from the supernatant and all adherent cells were collected, washed once with PBS, and digested with 0.25% trypsin–EDTA at 37 °C for 2–3 min. Following cell detachment, the reaction was terminated with complete culture medium. The cells were then centrifuged at 1000 rpm for 5 min, resuspended, and washed with PBS. Subsequently, the cells were resuspended in 100 μL of 1 × Binding Buffer, and 5 μL of Annexin V-FITC (C1062; Beyotime Biotechnology, Shanghai, China) and 5 μL of PI were added. The mixture was incubated at room temperature in the dark for 15 min, after which 400 μL of 1 × Binding Buffer was added. The sample was filtered through a 40 μm cell sieve, and at least 10,000 cells were collected for analysis. The flow cytometer (FACSCanto™ II; BD Biosciences, Franklin Lakes, NJ, USA) was excited at 488 nm and detected fluorescence signals at 530 nm (Annexin V-FITC, green) and >670 nm (PI, red). By analyzing the different fluorescence signals, the apoptosis rate of the cells was quantitatively assessed.

### 2.7. Research on Heavy Metal Mitigation

Previous research indicates that melanin exhibits strong chelating properties toward heavy metals, effectively mitigating their toxicity [11,38]. This investigation seeks to assess the potential alleviating impact of melanin derived from the *O. sinensis* TZ8-1 on heavy metals [39,40]. NHEK cells were cultured following established protocols and subjected to heavy metal exposure (Pb^2+^ and Cd^2+^). The experimental groups included a blank group (culture medium without cells), a blank control group (cells in fresh culture medium), a model group (cells in medium with 50 μM Pb^2+^ or 20 μM Cd^2+^), a normal control group (cells in medium with 50 μM Pb^2+^ or 20 μM Cd^2+^ plus EDTA) (E4884; Sigma-Aldrich, St. Louis, MO, USA), and a treatment group (cells in medium with 50 μM Pb^2+^or 20 μM Cd^2+^ and varying concentrations (0, 6.25, 12.5, 25, 50, 100, and 200 μg/mL) of melanin). Cell viability was assessed as previously described. Malondialdehyde (MDA) levels were measured using the thiobarbituric acid (TBA) method (S0131S; Beyotime Biotechnology, Shanghai, China), and glutathione (GSH) content was determined following Beyotime kit instructions (S0052; Beyotime Biotechnology, Shanghai, China) upon exposure to Pb^2+^ to evaluate the potential of TZ8-1 melanin in mitigating heavy metal effects. Data were obtained from three independent biological replicates, ensuring reproducibility of the mitigation effects.

### 2.8. Statistical Analysis

Data are presented as mean ± SD derived from three independent biological replicates per group. For in vitro studies, biological replicates corresponded to distinct experimental batches performed on different days. For the acute oral toxicity study (OECD 423), each mouse constituted a single biological replicate (*n* = 3 per group). Measurements within each replicate were performed in triplicate where technically feasible. Statistical significance was determined using one-way ANOVA followed by Tukey’s post hoc test in GraphPad Prism 10 (GraphPad Software, San Diego, CA, USA) and SPSS 27.0 (IBM Corp., Armonk, NY, USA) (*p* < 0.05). Origin 2024 (OriginLab Corporation, Northampton, MA, USA) was used for graphical representation.

## 3. Results and Discussion

### 3.1. TZ8-1 Identification of Melanin

#### 3.1.1. SEM Analysis

**Figure 1:**
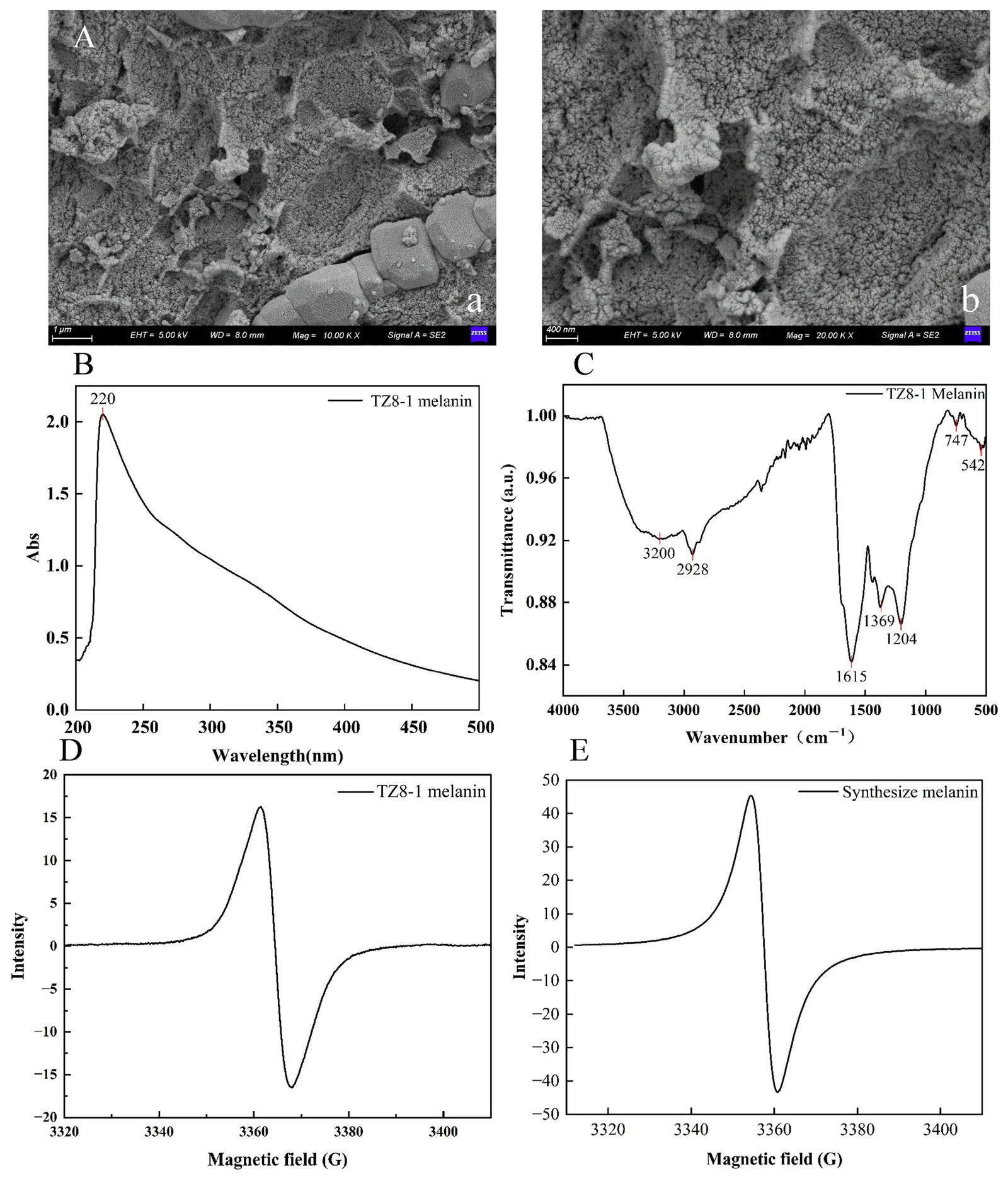
Structural identification of TZ8-1 melanin. (**A**) Scanning electron microscopy images at 10,000× (**a**) and 20,000× (**b**); (**B**) UV-Vis absorption spectrum; (**C**) FT-IR spectrum; (**D**) EPR spectrum of TZ8-1 melanin; and (**E**) EPR spectrum of synthetic melanin.

Scanning electron microscopy is an essential technique for characterizing pigment morphology. The morphology of melanin in TZ8-1 was analyzed using a scanning electron microscope (ZEISS Gemini SEM 500). The resulting electron microscope image displays an agglomerated microscopic morphology that is characterized by numerous fine particles that were aggregated (Figure 1A). The particle size is relatively uniform, the overall structure is loose, and numerous pores are present. The morphological characteristics of melanin closely resemble those of Streptomyces NTB42 [14], aligning with the features of natural melanin documented in existing studies. These morphological traits might be influenced by factors such as the sample preparation process, its intrinsic chemical composition, and intermolecular forces. Agglomeration might affect the physical and chemical properties of the sample, including specific surface area and adsorption performance. Additionally, the pore structure may be significant in application scenarios involving material transport and reaction interfaces.

#### 3.1.2. Elemental Analysis

The elemental composition of TZ8-1 melanin, including C, H, O, N, and S, was determined using an organic element analyzer. As shown in Table 1, eumelanin typically consists of 56.45% C, 3.15% H, 8.49% N, 31.82% O, and 0.09% S [41]. In contrast, melanin from *Inonotus hispidus* contained 42.55% C, 6.62% H, 5.31% N, 43.50% O, and 2.02% S [9]. According to research by Kazumasa Wakamatsu, eumelanin exhibited a sulfur-deficiency structure [42]. Previous studies have also indicated that eumelanin, a dark brown or black pigment, typically contains 6–9% nitrogen and 0–1% sulfur [11]. Through elemental analysis and comparison, TZ8-1 melanin aligns with the elemental composition characteristics of natural melanin, consistent with the recent report on *O. sinensis* melanin by Tong et al. which also identified a low but detectable sulfur content (1.20%) [3]. One-way analysis of variance (ANOVA) with Tukey’s post hoc multiple comparisons revealed no statistically significant differences in elemental contents between TZ8-1 melanin and melanin from other reported sources (*p* > 0.05).

#### 3.1.3. UV-Vis Analysis

The purified TZ8-1 melanin solution was analyzed by the entire spectrum using the ultraviolet–visible spectrophotometer UV-8000. The analysis revealed a maximum absorption wavelength at 220 nm (Figure 1B), beyond which the absorbance decreased gradually with increasing wavelength. Notably, no characteristic absorption peaks were detected across the spectrum, a feature consistent with the typical properties of melanin [9]. Specifically, the absence of characteristic absorption peaks in the 260–280 nm range indicated that TZ8-1 melanin did not contain nucleic acids, proteins, or lipids [16]. Comparative analysis with other melanin further contextualized the spectral characteristics of TZ8-1. For instance, the maximum absorption peaks of WAA melanin from wild black fungus and IH melanin were both located at 210 nm [9,44], while LIM-A melanin, NTB42 melanin, and synthetic melanin exhibited peaks at 215 nm, 218 nm, and 224 nm, respectively [14,45]. Additionally, melanin of *O. sinensis* showed a maximum absorption peak at 237 nm [3]. The absorption maximum of TZ8-1 melanin at 220 nm falls within the reported range for diverse melanin, supporting its identification.

#### 3.1.4. FT-IR Analysis

Infrared absorption spectroscopy serves as a crucial spectral feature for characterizing melanin, reflecting its chemical structure and functional group information [42]. The obtained spectrum of TZ8-1 melanin (Figure 1C) displayed several distinct absorption bands consistent with typical melanin features. In this spectrum, several major absorption peaks were observed at 3200, 2928, 1615, 1369, 1204, 747, and 542 cm^−1^. Specifically, the broad peak at 3200 cm^−1^ is attributed to O-H and N-H stretching vibrations [16,46] and was recognized as a characteristic spectral band of melanin. Notably, the absence of a distinct absorption band near 1720 cm^−1^ further corroborates the structural similarity to *O. sinensis* melanin characterized by Tong et al., indicating a lack of aliphatic ketones within the polymer matrix [3]. The absorption band at 2928 cm^−1^ indicates the presence of aliphatic C-H groups. The strong peak at 1615 cm^−1^ arises from aromatic C = C skeletal vibrations or the stretching vibration of quinoid carbonyl (C = O). The peak at 1369 cm^−1^ may originate from N-H bending or C-N stretching vibrations. The absorption at 1204 cm^−1^ is assigned to the C-OH stretching vibration of phenolic hydroxyl groups. The weak peaks at 747 cm^−1^ and 542 cm^−1^ correspond to out-of-plane C-H bending vibrations or substitution patterns on the aromatic ring. Overall, the FT-IR analysis reveals that TZ8-1 melanin exhibits typical characteristics of natural melanin, with key structural features including an aromatic skeleton, phenolic hydroxyl groups, aliphatic side chains, and amide moieties. These findings provide a reliable spectroscopic basis for the structural identification and subsequent application studies of this melanin [46].

#### 3.1.5. EPR Analysis

Electron paramagnetic resonance (EPR) is used to detect paramagnetic centers, specifically unpaired spin states, in a substance [42]. The EPR spectrum of TZ8-1 melanin demonstrates a slightly asymmetrical single peak, closely resembling that of synthetic melanin and aligning with the morphological characteristics observed in previous studies, such as KUC3009 melanin derived from resin [43], TPBM melanin sourced from rhizobia [13], and Apis melanin from *Haematococcus pluvialis* [38]. The presence of this signal confirms unpaired electrons in TZ8-1 melanin. Notably, the absence of distinct hyperfine structure associated with unpaired electrons and nitrogen nuclei suggests that brown melanin is absent in TZ8-1 melanin [15,43,47,48]. Research indicate that the EPR signal of eumelanin arises from quinone radicals [49], typically exhibiting a slightly asymmetric spectrum with G-values ranging from 2.003 to 2.006 [50]. The G-factor of TZ8-1 melanin is measured at 2.005, which is in close agreement with the value of 2.00508 reported by Tong et al. [3] for *O. sinensis* melanin, consistent with the EPR characteristics associated with eumelanin [47,48].

#### 3.1.6. PY-GC/MS Analysis

Pyrolysis–gas chromatography/mass spectrometry (PY-GC/MS) represents a widely employed and efficient technique for the characterization of melanin [19,51]. This method involves heating the polymer sample to convert it into a mixture of decomposition products, which are subsequently analyzed using GC/MS, thereby largely reflecting the original structure of the sample. The pyrolysis diagram of TZ8-1 melanin is presented in the Supplementary Materials (Table S1). The result indicated that a total of 50 compounds were identified in the pyrolysis product of TZ8-1 melanin, predominantly including benzene, phenol, pyridine, furan, indole, and pyrrole, with benzene and its derivatives being the most prevalent. Existing studies have established that the primary products of eumelanin pyrolysis consist of benzene, phenol, pyrrole, indole, and their derivatives [52], a diagnostic profile that closely matches the pyrolytic fingerprint of *O. sinensis* melanin reported by Tong et al. [3]. Additionally, pyridine was detected in the analysis. The decomposition of isoquinoline, a marker product of browning melanin, could yield pyridine and its alkyl derivatives [52]. However, findings from EPR studies indicated that browning melanin was not present. The detection of pyridine compounds in TZ8-1 melanin may account for the low protein content in the sample [20,53]. Furthermore, furans were identified among the pyrolysis products of TZ8-1 melanin, aligning with a previous study on the pyrolysis products of natural melanin and specifically corroborating the findings of Tong et al. [3], who identified furan as a characteristic component of *O. sinensis* melanin. In conclusion, the melanin in TZ8-1 exhibits characteristics consistent with eumelanin, corroborating the findings from FT-IR and EPR analyses and validating the structural congruence with the recently characterized *O. sinensis* melanin via Py-GC/MS [3].

### 3.2. Safety Inspection

#### 3.2.1. Cytotoxicity

**Figure 2:**
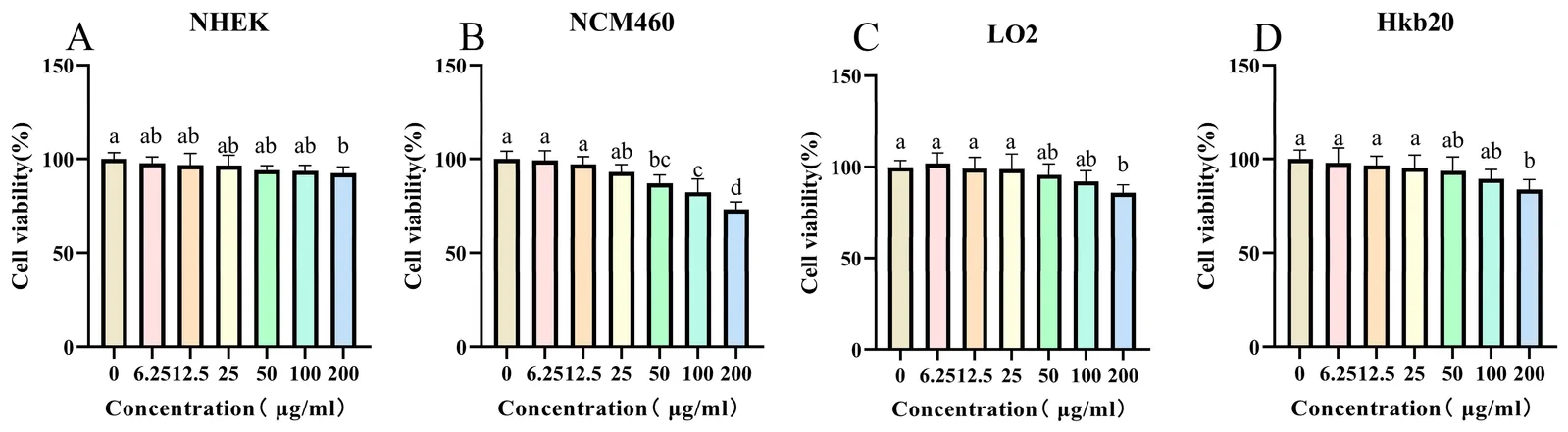
Cytotoxicity of TZ8-1 melanin against four normal human cell lines. Cell viability was measured by CCK-8 assay after 48 h of exposure to TZ8-1 melanin (0–200 μg/mL). (**A**) NHEK, (**B**) NCM460, (**C**) LO2, and (**D**) Hkb20. Data are presented as mean ± SD (*n* = 3). The coefficients of variation (CVs) for all tested groups were consistently low (ranging from 2.1% to 8.9%), indicating high experimental reproducibility. Bars sharing the same lowercase letter indicate no significant difference *(p* > 0.05), while different letters denote a significant difference (*p* < 0.05). Statistical comparisons were performed using one-way ANOVA with Tukey’s post hoc test.

The viability of four cell types (NHEK, LO2, Hkb20, and NCM460) exposed to varying concentrations of TZ8-1 melanin was assessed using the CCK-8 method to investigate the cytotoxicity of TZ8-1 melanin [29,54]. The results, depicted in Figure 2, revealed dose-dependent reductions in cell survival rates across all cell types following melanin exposure. As the melanin concentration increased, cell survival rates progressively declined, with respective half-maximal inhibitory concentrations (IC50) of 22.839 mg/mL, 3.125 mg/mL, 2.653 mg/mL, and 1.086 mg/mL. Upon treatment of NHEK, Hkb20, and LO_2_ cells with TZ8-1 melanin, there were no significant differences in cell viability observed within the 0–100 μg/mL concentration range compared to the control group (0 μg/mL) (*p* > 0.05). These findings suggested that treatment within this concentration range did not induce notable toxicity in NHEK, Hkb20, and LO2 cells [24]. However, at 200 μg/mL, cell survival rates decreased by 7.46%, 16.14%, and 14.09%, respectively. This indicates a dose-dependent decrease in viability of NHEK and NCM460 cells with increasing treatment concentrations. Compared to the control group, the viability of cells in 50 μg/mL treatment group decreased by 12.78%. When the concentration was further increased to 100 μg/mL and 200 μg/mL, the inhibitory effect intensified, resulting in cell survival rate decrease by 17.60% and 26.83%, respectively. These findings indicated that NCM460 cells exhibited a high sensitivity to TZ8-1 melanin treatment. A concentration of 50 μg/mL can induce significant cytotoxicity, with higher concentrations leading to a more pronounced inhibitory effect. The results also suggest that TZ8-1 melanin has a relatively low cytotoxic effect on four cell types: skin, intestinal, liver, and kidney, as indicated by IC50 values exceeding 1 mg/mL.

#### 3.2.2. Acute Oral Test

Within 14 d following administration, no fatalities were recorded in any of three groups of experimental mice, and the systematic behavior assessment revealed no adverse reactions [27,32]. The weight changes of the experimental mice are presented in Table 2. The findings indicated that after oral administration of melanin suspension to C57 mice, the animals demonstrated healthy growth, without evident sign of toxicity. A univariate analysis was conducted on the body weight of the mice prior to administration. No statistically significant differences in initial body weight were observed between groups (*p* > 0.05), supporting the validity of subsequent comparative analyses. Notably, within each time point, no statistically significant differences in body weight were observed among the groups at 1 and 7 d post-treatment. Furthermore, no significant intragroup variations in body weight were detected across different time points (days 1, 7, and 14) within the same treatment group. However, the body weight in the 5000 mg/kg group was significantly lower than in the controls at 14 d (*p* < 0.05). In contrast, the body weight in the 2000 mg/kg group remained comparable to all other groups.

#### 3.2.3. Blood Test

Blood samples containing anticoagulants were utilized for routine analysis in this study [27] (Table 3). There were no statistically significant differences (*p* > 0.05) between the 2000 mg/kg and control groups regarding white blood cell (WBC) and red blood cell (RBC) counts (*p* > 0.05), suggesting an absence of adverse effects on the hematopoietic system at this dosage. This finding suggested that the drug did not significantly affect the hematopoietic system of mice at this dosage. In the 5000 mg/kg administration group, a significant increase in WBC levels was noted 1 d post-administration (*p* < 0.05); however, no statistically significant differences were found in RBC levels at any time point relative to the control group (*p* > 0.05). These findings imply that TZ8-1 melanin does not elicit significant toxicity toward erythropoiesis or disrupt RBC homeostasis, even at higher doses [31]. The observed increase in WBCs at the 5000 mg/kg dose may reflect a mild immunostimulatory effect. Such a response could be indicative of hematopoietic activation, possibly through the promotion of hematopoietic stem cell proliferation or enhanced differentiation and activity of immune cells, as suggested by previous studies [31]. This phenomenon is often associated with compounds that modulate innate immunity or induce transient inflammatory stimuli. However, the absence of RBC alterations underscores the specificity of this effect, confining it largely to the myeloid lineage without compromising erythroid integrity. From a toxicological perspective, the dissociation between WBC elevation and RBC stability is noteworthy. It suggests that TZ8-1 melanin may interact with immune signaling pathways without inducing broad myelosuppression or hemolytic damage.

#### 3.2.4. Blood Biochemical Testing

Serum biochemical analysis was performed to evaluate the hepatorenal toxicity of TZ8-1 melanin in mice after acute oral exposure [30] (Table S2). The results indicated that TZ8-1 melanin did not exhibit clear signs of toxicity at the low dose but showed potential hepatotoxic effects at the high dose, with the toxicological threshold lying between 2000 mg/kg and 5000 mg/kg. At the 2000 mg/kg dose, all liver and kidney function parameters showed no significant changes, establishing this dose as the no-observed-adverse-effect level (NOAEL), which suggested a high acute safety profile at this dosage. In the 5000 mg/kg group, signs of potential hepatotoxicity were observed. The sharp increase in AST levels (*p* = 0.054) was of toxicological importance, as it typically indicates hepatocellular stress or damage, potentially involving organelles such as mitochondria. Although the increase in ALT was not statistically significant, its concurrent upward trend with AST strongly suggested that the 5000 mg/kg dose approached the threshold for toxicity. In contrast, renal function indicators (CRE and BUN) remained stable across all dose groups, indicating that the kidneys were not the primary target organ of acute TZ8-1 melanin toxicity. In conclusion, TZ8-1 melanin exhibited a wide safety window in this acute exposure study; however, the early signs of liver injury induced by the high dose warrant further investigation through histopathological examination and subchronic toxicity studies [55,56].

#### 3.2.5. Histopathological Examination

**Figure 3:**
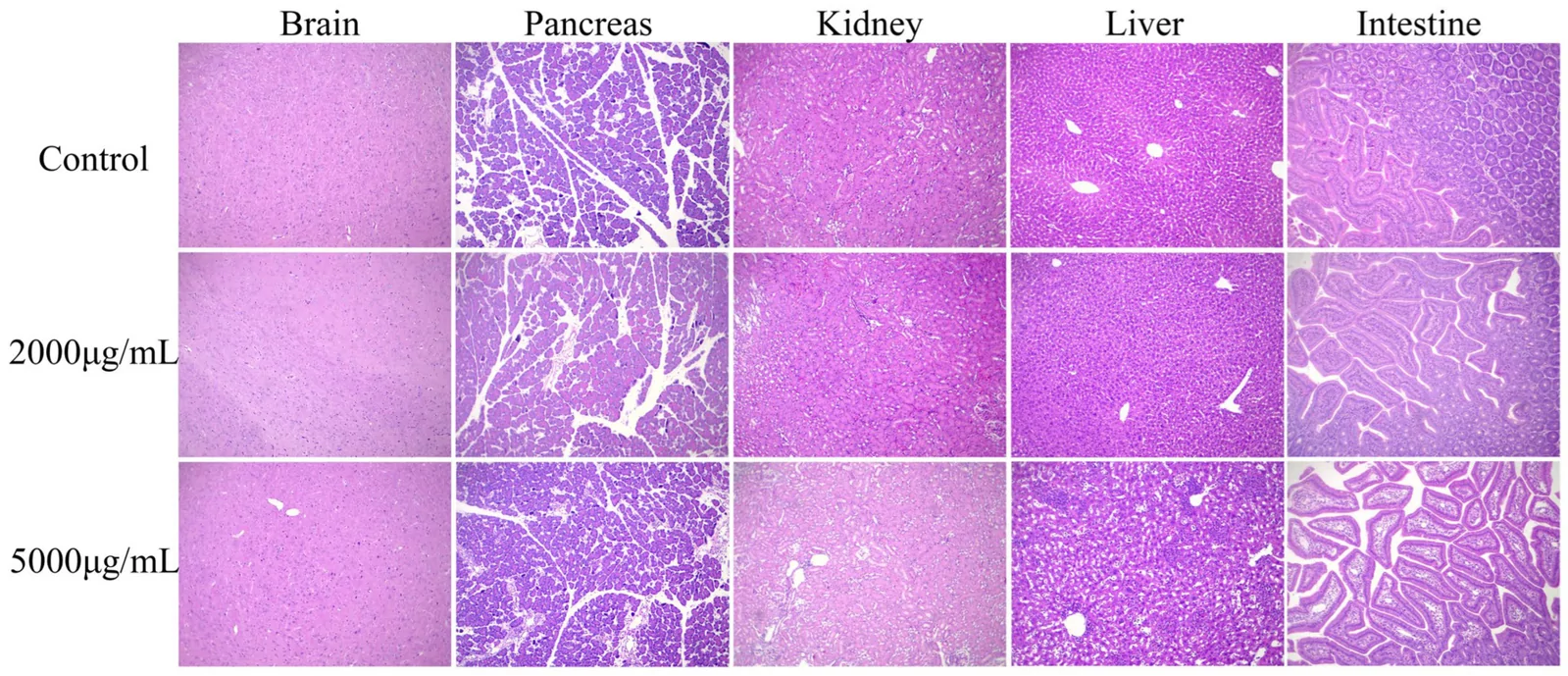
Histopathological examination of major organs after TZ8-1 melanin treatment. Representative H&E-stained sections from the brain, pancreas, kidney, liver, and intestine. Images are arranged in rows: control, 2000 μg/mL, and 5000 μg/mL treatment groups (top to bottom).

This study conducted a comprehensive evaluation of the acute oral toxicity of TZ8-1 melanin by integrating histopathological examination with serum biochemical parameters [27]. Histopathological analysis revealed (Figure 3) no pathological alterations in the brain, pancreas, kidney, or intestinal tissues across all experimental groups. In contrast, liver sections from the high-dose group exhibited significant lesions, characterized by hepatocellular vacuolation, disorganized cord architecture, and mild inflammatory infiltration. These pathological findings provided morphological correlates for the observed elevation in plasma AST levels. The presence of vacuolation served as a critical indicator of intracellular edema or lipid accumulation, suggesting potential mitochondrial dysfunction or disrupted energy metabolism. This observation aligns with the marked increase in AST, a mitochondrial enzyme, indicating that mitochondrial impairment likely represented the initial cellular damage in hepatocytes. Critically, the 2000 mg/kg group showed no such pathological changes, establishing this dose as the NOAEL [31,56]. The integrated histopathological and biochemical analyses demonstrated that TZ8-1 melanin exhibits a wide safety margin at 2000 mg/kg, with the liver emerging as its primary target organ at 5000 mg/kg, thereby validating previous findings [56]. Future investigations should focus on elucidating the mechanisms underlying this mitochondrial-centric hepatotoxicity, potentially involving oxidative stress pathways [30,31].

### 3.3. Light Protection

#### 3.3.1. Cell Survival Rate Under UVB Irradiation

**Figure 4:**
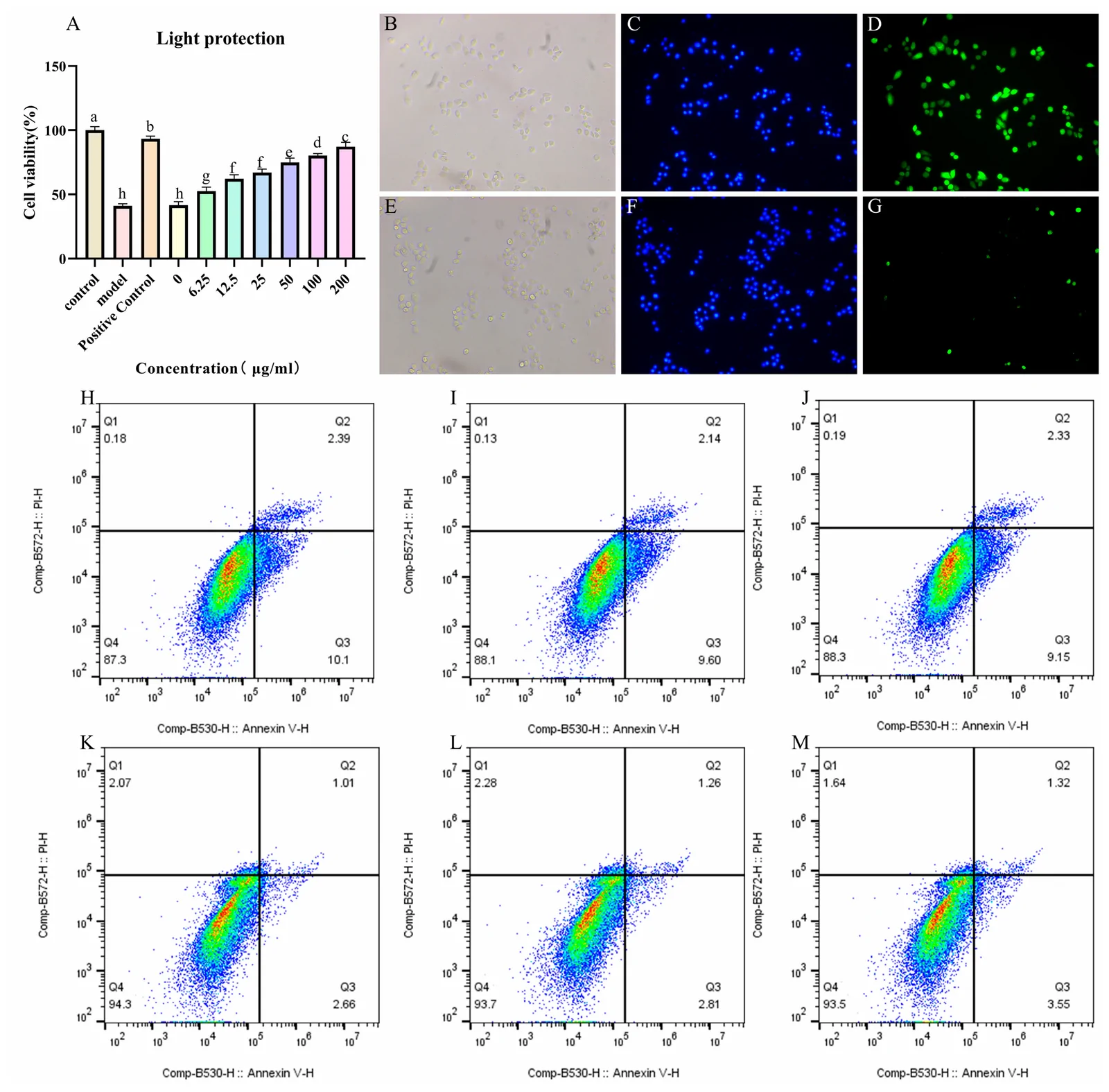
Photoprotective efficacy of TZ8-1 melanin against UV-induced damage. (**A**) Cell viability assessed by CCK-8 assay post-UV irradiation. Data are mean ± SD (*n* = 3). Coefficients of variation (CVs) were all below 8%, indicating high reliability of the assay. Different lowercase letters indicate significant differences (*p* < 0.05, one-way ANOVA with Tukey’s test). (**B**–**G**) Representative fluorescence microscopy images. (**B**–**D**) Control group; (**E**–**G**) 200 μg/mL TZ8-1 treatment. Blue: nuclear staining (DAPI); Green: ROS fluorescence. (**H**–**M**) Flow cytometric analysis of apoptosis. (**H**–**J**) Control; (**K**–**M**) 200 μg/mL TZ8-1 treatment. In dot plots, color gradient (blue to red) indicates cell density; quadrants (Q1–Q4) classify cell status (e.g., live, early/late apoptotic).

The CCK-8 method was employed to assess the protective effect of TZ8-1 melanin on cells subjected to ultraviolet irradiation, thereby elucidating its potential anti-radiation properties [57]. The results are illustrated in Figure 4A. The observed decline in cell viability within the model group confirmed the efficacy of the experimental modeling. Analysis of the treatment groups with varying concentrations of TZ8-1 melanin (0, 6.25, 12.5, 25, 50, 100, and 200 μg/mL) revealed a clear concentration-dependent increase in cell survival rates following ultraviolet exposure as the melanin concentration escalated from 0, 6.25 μg/mL to 200 μg/mL. At concentrations of 50 and 100 μg/mL, the protective effect became more pronounced, with cell survival rates approaching those of the control group. When the concentration was 200 μg/mL, the cell survival rate increased to 87.25%, which proved that TZ8-1 melanin has great protective potential against UVB-damaged cells [58]. The distinct letter markings in Figure 4 indicate statistical differences among the groups. The survival rates across each concentration group of TZ8-1, compared to the model group, are statistically significant, further corroborating the concentration dependent protective effect.

#### 3.3.2. ROS Generation

Studies have demonstrated that melanin possesses photoprotective properties by inhibiting the production of reactive oxygen species (ROS) within cells [59]. UVB typically induces ROS generation, which could lead to the rupture of the outer mitochondrial membrane and subsequently result in apoptosis [60]. In this experiment, the DCFH-DA fluorescence probe method was employed to assess ROS levels in Normal Human Epidermal Keratinocytes (NHEKs) subjected to UVB irradiation. When excited at a specific wavelength of 488 nm, the presence of ROS causes the cells to emit green fluorescence. Following melanin treatment and subsequent ROS detection and analysis, it was observed that the model group exhibited significant green fluorescence after UVB exposure (Figure 4B–G). In contrast, 200 μg/mL TZ8-1 melanin treatment group shows a marked reduction in green fluorescence compared to the model group, indicating that the ROS content in the melanin treatment group is considerably lower than that in the control group. Therefore, melanin derived from TZ8-1 demonstrates the ability to inhibit ROS production [61,62].

#### 3.3.3. Apoptosis Experiment

Based on the flow cytometric analysis of the apoptosis experiment in Figure 4H–M (using Annexin V-FITC/PI double staining) [63], the results demonstrate that TZ8-1 melanin significantly inhibits UVB-induced apoptosis: the control-group cell distribution shows 88.3% viable cells (Annexin V^−^/PI^−^, Q4 quadrant), 9.15% early apoptotic cells (Annexin V^+^/PI^−^, Q3 quadrant), 2.33% late apoptotic/necrotic cells (Annexin V^+^/PI^+^, Q2 quadrant), and 0.19% other dead cells (Annexin V^−^/PI^+^, Q1 quadrant). In contrast, after treatment with TZ8-1 melanin, the experimental group exhibited a significant increase in viable cells to 94.3% (*p* < 0.01), while early and late apoptotic/necrotic cells decreased to 2.68% and 1.01%, respectively. The Q1 quadrant shows a slight increase to 2.07%, but this proportion remains low (<3%) and may be associated with non-specific necrosis or autophagic death, not affecting the overall conclusion. These findings confirm that TZ8-1 melanin exerts its protective effects through a dual mechanism: on the one hand, it suppresses the initiation of early apoptotic signals (such as mitochondrial membrane potential disruption or caspase cascade activation) [60], and on the other hand, it reduces damage caused by loss of membrane integrity at later stages, thereby significantly enhancing cell survival [64,65]. In summary, TZ8-1 melanin effectively mitigates UVB radiation-induced apoptosis, with its antioxidant properties and free radical scavenging capacity likely serving as key mechanisms, providing experimental evidence for its potential in photoprotection applications. TZ8-1 melanin demonstrated significant, concentration-dependent protection against UVB irradiation. It markedly enhanced cell viability, concurrently suppressed intracellular ROS generation, and effectively inhibited UV-induced apoptosis. These results collectively affirm its potent photoprotective efficacy through antioxidative and anti-apoptotic mechanisms.

### 3.4. Heavy Metal Mitigation

**Figure 5:**
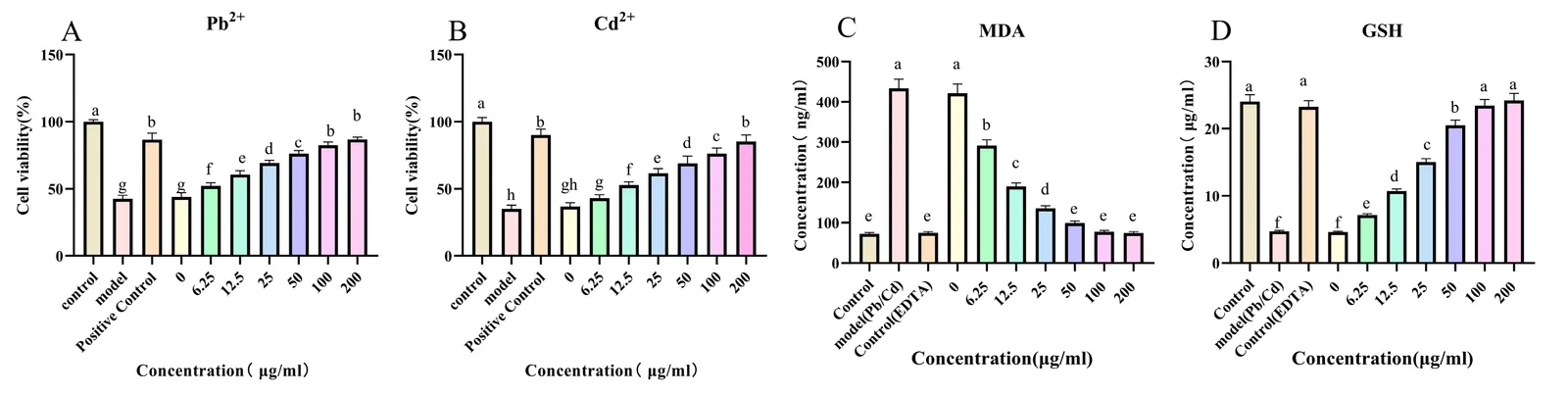
TZ8-1 melanin mitigates Pb^2+^- and Cd^2+^-induced cytotoxicity and oxidative stress in NHEK cells. (**A**,**B**) Cell viability of NHEK cells exposed to Pb^2+^ (**A**) or Cd^2+^ (**B**), assessed by CCK-8 assay. (**C**,**D**) Oxidative stress markers in Pb^2+^-exposed cells, including MDA content (**C**) and GSH content (**D**). Data are mean ± SD (*n* = 3). Coefficients of variation (CVs) were all below 9%, indicating high reliability of the assay. Bars with different lowercase letters indicate significant differences (*p* < 0.05, one-way ANOVA with Tukey’s post hoc test).

This experiment investigated the cytotoxic effects of the heavy metals Pb^2+^ and Cd^2+^ on NHEK cells, as well as the protective role of TZ8-1 melanin, with underlying mechanisms elucidated through multi-dimensional analyses of cell survival rates and oxidative stress markers (MDA and GSH). Initial results confirm the cytotoxicity of Pb^2+^ and Cd^2+^ (Figure 5A,B): compared to the blank group, the model group exposed to these metals exhibited a significant reduction in cell survival rate (*p* < 0.01), indicating direct toxic effects on cellular viability. The positive control group (EDTA) also shows lower survival rates than the blank group, further validating the experimental model. Subsequent treatment with TZ8-1 melanin at graded concentrations (0–200 μg/mL) induced a dose-dependent increase in cell survival rates. At 200 μg/mL, survival rates for the Pb^2+^ and Cd^2+^ groups reached 86.82% and 85.14%, respectively (Figure 5A,B), suggesting a concentration-dependent protective effect. This protection may stem from mechanisms such as heavy metal ion chelation or activation of endogenous defense pathways [66]. Analysis of oxidative stress markers revealed that Pb^2+^ exposure significantly elevated MDA levels (*p* < 0.01), reflecting increased lipid peroxidation [67]. Following TZ8-1 treatment, MDA levels decrease significantly in a concentration-dependent manner (*p* < 0.05), indicating suppression of membrane lipid peroxidation chain reactions—possibly via free radical scavenging or inhibition of lipoxygenase activity. Conversely, the model group displayed markedly lower GSH levels than the blank group (*p* < 0.01), suggesting heavy metals impair GSH synthesis or accelerate its consumption. TZ8-1 treatment restores GSH levels in a concentration-correlated fashion (*p* < 0.05), potentially by upregulating glutathione synthase (γ-glutamylcysteine synthase, γ-GCS) or inhibiting GSH oxidation to GSSG. In summary, TZ8-1 melanin mitigates Pb^2+^ /Cd^2+^ toxicity through a dual antioxidant mechanism: it directly attenuates lipid peroxidation and enhances endogenous antioxidant defenses by elevating GSH levels. Notably, its protective efficacy strengthens with increasing concentration, supporting its potential as a heavy metal detoxification candidate [68]. Future studies should explore its binding kinetics with heavy metals and interactions with target proteins (e.g., metallothionein) [67,69,70,71].

## 4. Conclusions

This study purified TZ8-1 using organic solvent washing and characterized its structure via SEM, EA, UV-Vis, FT-IR, EPR, and PY-GC/MS. Structural analysis revealed that TZ8-1 conforms to the basic characteristics of natural melanin, with EPR and EA indicating it aligns more closely with eumelanin properties. Safety evaluation shows low cytotoxicity of TZ8-1 toward four human normal cell lines in cytotoxicity assays; acute oral toxicity testing reveals no mortality or behavioral abnormalities at any dose, confirming a high safety profile. The non-significant elevations in serum ALT and AST in the high-dose group (*p* > 0.05) suggest potential liver stress at this level, justifying further studies on long-term hepatic effects. Biologically, TZ8-1 exhibited potent photoprotective effects against UVB radiation, significantly enhancing cell viability, suppressing ROS production, and reducing apoptosis from 11.48% to 3.69%, a mechanism likely involving phenolic hydroxyl group-mediated free radical scavenging. Heavy metal-alleviation assays indicated that under heavy metal exposure, TZ8-1 effectively mitigated cell death, increased survival rates, reduced lipid peroxidation product MDA, and elevated antioxidant GSH levels, confirming its protective role via chelating heavy metal ions and enhancing cellular antioxidant defenses. Notably, TZ8-1’s photoprotective efficiency (87.25%) approaches that of vitamin C (93.42%) [72], while its heavy metal-alleviation performance matches EDTA, highlighting its potential as a natural antioxidant alternative. This study has some limitations. The bioactivity and toxicity data were derived solely from in vitro cellular assays and a 14-day acute toxicity test, lacking in vivo validation and long-term safety profiles. While characterized as eumelanin, the specific biosynthetic pathway (DOPA vs. DHN) and precise molecular weight remain unclear. Additionally, the photoprotection tests were restricted to UVB, and heavy metal mitigation was only assessed for Pb^2+^ and Cd^2+^. Future research should explore in vivo toxicological mechanisms, investigate nano-modifications to improve bioavailability, and expand its applications to antitumor therapy and drug delivery [73,74,75].

## Figures and Tables

**Table 1:** Elemental composition of different melanin types.

Types of Melanin	C (%)	H (%)	O (%)	N (%)	S (%)	Reference
TZ8-1 melanin	59.89	5.80	26.09	7.49	0.80	
*O. sinensis* melanin	50.59	6.18	33.90	8.19	1.20	[3]
IH melanin	42.55	6.62	43.50	5.31	2.02	[9]
Synthetic melanin	56.45	3.15	31.82	8.49	0.09	[41]
*A. resinae* KUC3009 melanin	52.70	4.60	34.20	6.90	0.80	[43]

Table note: Data are expressed as mean ± SD for TZ8-1 melanin (3 independent parallel measurements, relative standard deviation < 2% for all elements); values for other melanin types are cited from the published literature as single representative measurements.

**Table 2:** The average weight change of mice 14 d after administration.

Days	Control	2000 mg/kg	5000 mg/kg
1 d	30.35 ± 5.62	27.22 ± 0.50	24.97 ± 4.28
7 d	31.33 ± 4.18	28.00 ± 1.15	26.70 ± 3.30
14 d	31.87 ± 3.45	29.00 ± 0.79	25.20 ± 2.21 *

Table note: Data are expressed as mean ± SD (*n* = 3 per group). * *p* < 0.05 compared with the control group at the same time point. No significant intragroup differences were detected across different time points (days 1, 7, and 14) within the same treatment group.

**Table 3:** Hematological analysis of acute oral test.

Types	Control	2000 mg/kg	5000 mg/kg
WBC (×10^9^/L)	4.417 ± 0.500	4.620 ± 0.383	11.493 ± 6.491 *
RBC (×10^12^/L)	10.733 ± 0.088	10.483 ± 0.658	9.113 ± 1.436

Table note: Data are expressed as mean ± SD ( *n* = 3 per group). * *p* < 0.05 versus the control group at the 14 d endpoint. WBC = white blood cell; RBC = red blood cell.

## Data Availability

The original contributions presented in this study are included in the article. Further inquiries can be directed to the corresponding authors.

## Supplementary Materials

The following supporting information can be downloaded at https://www.mdpi.com/article/10.3390/jof12080613/s1, Table S1: Thermal decomposition gas chromatography and mass spectrometry combined analysis table; Table S2: Biochemical analysis of acute oral experiments.

## Abbreviations

UV-Vis, ultraviolet–visible spectroscopy; FT-IR, Fourier-transform infrared spectroscopy; EA, elemental analysis; Py-GC/MS, pyrolysis gas chromatography/mass spectrometry; ROS, reactive oxygen species; MDA, malondialdehyde; GSH, glutathione; NHEKs, Normal Human Epidermal Keratinocytes; LO2, human normal liver cell line; NCM460, normal colon mucosal epithelial cells; Pb^2+^, lead ion; Cd^2+^, cadmium ion.
