# Supplementary material for: Toxicological Assessment and Dual Protective Roles of Ophiocordyceps sinensis Melanin: Photoprotection and Heavy Metal Detoxification
Source: J Fungi (Basel). 2026 Aug 15;12(8):613. doi: 10.3390/jof12080613 (PMC13514701; doi:10.3390/jof12080613)
Supplement: Supplementary file 1 [file jof-12-00613-s001.zip › jof-4402702-supplementary.pdf]

Supplementary Material

Table S1 Thermal decomposition gas chromatography and mass spectrometry combined analysis table

Peak Signal	Retention time	Peak area	Structure	Peak Signal	Retention time	Peak area	Structure
1	2.655	1.87	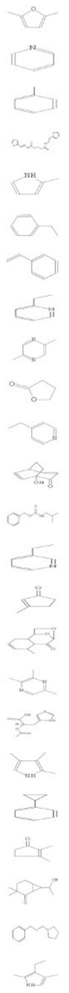	26	7.780	1.07	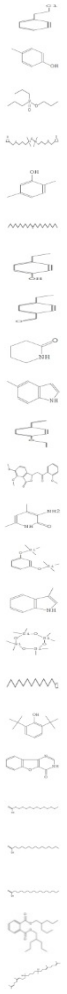
2	2.930	2.03		27	7.910	6.98	
3	3.140	18.89		28	8.050	2.30	
4	3.525	0.42		29	8.290	1.44	
5	4.020	2.13		30	8.825	1.36	
6	4.170	8.15		31	8.995	0.56	
7	4.535	6.68		32	9.190	3.02	
8	4.715	0.79		33	9.435	0.41	
9	4.800	0.92		34	9.490	0.44	
10	4.880	1.46		35	9.795	0.64	
11	5.120	3.50		36	10.310	1.29	
12	5.280	1.50		37	10.505	0.67	
13	5.390	1.08		38	10.610	0.72	
14	5.495	0.55		39	11.100	0.85	
15	5.610	1.28		40	12.110	0.65	
16	5.990	1.06		41	12.245	0.41	
17	6.105	0.41		42	12.750	0.36	
18	6.350	0.37		43	13.450	1.10	
19	6.430	0.84		44	15.905	1.17	
20	6.495	0.85		45	16.425	1.10	
21	6.650	2.97		46	17.460	0.58	
23	6.835	0.96		48	18.465	2.88	
24	6.920	0.55		49	23.340	0.55	
25	7.585	0.63		50	25.670	7.03	

Table S2 Biochemical analysis of acute oral experiments

Group of mice	ALT（U/L）		AST（U/L）		CRE（μmol/L）		BUN（mmol/L）	
	Plasma level	p-value	Plasma level	p-value	Plasma level	p-value	Plasma level	p-value
control	50.33±26.08		193.00±8.00		40.33±16.44		6.06±0.91	
2000mg/kg	50.67±24.11	0.993	170.67±38.55	0.792	29.67±2.08	0.548	5.99±0.98	0.929
5000mg/kg	111.00±62.95	0.125	386.33±167.34	0.054	69.33±31.47	0.134	5.37±1.00	0.414
Reference range	28-132		59-247		27-88		5-10.3	
